# Circadian Typology and Personality Dimensions of Croatian Students of Health-Related University Majors

**DOI:** 10.3390/ijerph17134794

**Published:** 2020-07-03

**Authors:** Jakov Milić, Iva Milić Vranješ, Ivana Krajina, Marija Heffer, Ivana Škrlec

**Affiliations:** 1 Faculty of Medicine Osijek, Josip Juraj Strossmayer University of Osijek, 31000 Osijek, Croatia; miliciva@yahoo.com (I.M.V.); krajina.ivana91@gmail.com (I.K.); mheffer@mefos.hr (M.H.); 2 Clinic for Gynecology and Obstetrics, Osijek University Hospital, 31000 Osijek, Croatia; 3 Department for Dermatology and Venereology, Osijek University Hospital, 31000 Osijek, Croatia; 4 Histology, Genetics, Cellular, and Molecular Biology Laboratory, Faculty of Dental Medicine and Health, Josip Juraj Strossmayer University of Osijek, 31000 Osijek, Croatia

**Keywords:** morningness-eveningness, chronobiology, medical students, university students, Big-Five

## Abstract

The aim of this study was to determine the relationship between circadian preferences and personality dimensions among 712 students of three different majors from the Faculty of Medicine, Osijek: medical students (MD), nursing students (RN) and medical laboratory diagnostics students (MLD). For the measurement of personality dimensions, the IPIP50 Big-Five questionnaire was used. The circadian preference of students was assessed using the reduced morningness-eveningness questionnaire (rMEQ). Several significant results were observed and there was a significant difference in circadian preference among the three tested groups, with RN students scoring highest on the morningness scale and MLD students scoring the lowest. RN students scored significantly higher on agreeableness and conscientiousness than the other two groups. On the other hand, MD students scored higher on intellect than the MLD students. MLD students scored the lowest on emotional stability scales. Morning type students had higher conscientiousness and emotional stability scores. These results imply that circadian preference and personality traits are crucial elements of medical professionals’ wellbeing. With this paper, we would like to raise awareness about common personality traits and adherence to certain circadian orientations in medical professionals as a motivation to introduce a more flexible view towards strict time and task divisions in everyday practice.

## 1. Introduction

Students differ in their biological rhythms, such as wake time, bedtime or time at which they feel at their best [1,2]. Circadian rhythms show many physiological processes with the most prominent circadian rhythms in mammals being sleep and waking. Physiological rhythms are also seen in core body temperature, secretion of hormones such as cortisol and melatonin and activity of many organ systems. The suprachiasmatic nucleus (SCN) is the central pacemaker or the master clock in mammals [3]. Circadian preference shows substantial differences in biological and behavioral parameters [1,2]. In the concept of circadian preference, individuals may be classified in one of three chronotypes: morning, neither and evening type [4]. About 60% of the adult population is classified as neither type, while 40% are in one of the two extreme groups [1]. Studies show that most medical students (52.6% to 67.2%) are neither type [5,6,7], which is similar to the adult population.

For the understanding of the relationships between circadian rhythms and behavior, in the past, research on personality has been conducted using different questionnaires and morningness measures [8,9]. The International Personality Item Pool Five-Factor model (IPIP Big-Five) has five broad dimensions: extraversion, agreeableness, conscientiousness, emotional stability and intellect [10]. The dimensions of the IPIP Big-Five model have proved crucial in predicting achievements in life, especially educational and academic success [11]. Previous studies have reported an association between personality dimensions and a wide range of mental problems. Lower emotional stability is associated with anxiety, depression and other negative emotions [11,12,13,14,15,16], while higher extroversion protects against depressive symptoms [16,17]. Circadian preference has been considered a potential intervening factor in affective and other minor psychiatric disorders [18] and chronotype has been found to correlate with some personality dimensions [19]. Studies show that evening preference is associated with impulsivity, more depressive symptoms and possible psychiatric diseases [18,19,20,21]. In the literature are many inconsistent results regarding the correlation between circadian preference and personality dimensions. Meta-analyses results show that morningness is related to conscientiousness and, in a smaller degree, to agreeableness [19,22,23,24], while extraversion and intellect are related to eveningness [22,23]. Low emotional stability scores, associated with evening types, might indicate a tendency for frustration, emotional instability and depression [12,16]. Jackson and Gerard proposed that conscientiousness is the personality dimension that best distinguishes diurnal types [25]. The link between circadian rhythm and personality is a potential common neurobiological model because serotonin is implicated in both control of circadian rhythm as well as an individual’s psychological state. Serotonergic inputs to the SCN adjust the entrainment of circadian rhythms to light and also regulate activity-induced shifts in the circadian rhythm [26,27] and personality [28].

The majority of the conducted research is based on a single student population, such as medical students or psychology students, and all the studies analyze the influence of personality dimensions on circadian preference or vice versa. No studies compare personality dimensions and morningness-eveningness orientation between physicians, nurses and laboratory assistants. Circadian preferences and personality differences, which could relate to students’ future jobs, might be present between students from distinct study programs as early as in student age. Circadian preferences change with age, while personality dimensions are relatively stable throughout time [29]. Different types of people choose different study programs that differ from each other due to workload and schedule. It is considered that medical students have more study materials and less free time. This information raises interest into whether medical students have morning preferences or are more evening-orientated, compared to other study programs, and how medical professionals differ in personality dimensions. Students’ lectures are usually held in the morning and this could lead to sleep deprivation. Previous research at our faculty has shown that over 50% of students are depressed [16]. Determining students’ circadian preferences might help us assess their schedule, whether it fits their habits or should be changed. Furthermore, assessing personality dimensions between different majors could be useful in the evaluation of which personality traits contribute to the later chronotype and whether those personality traits might contribute to affective disorders. It is well known that neuroticism (e.g., low emotional stability) leads to affective disorders [15,18]. Medical students (MD), nursing students (RN) and medical laboratory diagnostics students (MLD) were analyzed separately because these three groups differ in many respects. We expect MD to be more ambitious and emotionally more stable than other students. RN would be more sensitive and perhaps more extroverted. This difference can be reflected in the circadian preferences and, consequently, in possible affective disorders.

The aim of the study was to investigate the relationship between the students’ major and personality dimensions on the one hand and circadian preferences on the other. Furthermore, the aim was to study the relationship between circadian preference and personality dimensions in students of different majors. To the authors’ knowledge, rMEQ has not been used in research in Croatia to this point. The further aim was to assess the psychometric reliability of the instrument in the population of students from biomedical studies. In this case, medical doctors, nursing students and medical laboratory diagnostic students from the Faculty of Medicine, Osijek, Croatia.

## 2. Participants and Methods

### 2.1. Participants

A cross-sectional study was conducted from 7 January to 15 June 2016, on 944 students from the Faculty of Medicine Osijek, Croatia. Out of these, 712 students (201 males) completed the survey (the response rate was 75.43%). All participants were anonymous and unpaid volunteers, asked to fill in the questionnaire after lectures in person and all gave their written informed consent before inclusion in the study. All students enrolled in 2015/2016 were involved in the research, which includes students of all study years. Inclusion criteria: medical, nursing and medical laboratory diagnostics students present at class on the day of data collection. There were no specific exclusion criteria. The average age of the participants was 22.93 ± 5.88. Participants were divided into three groups, based on their major: (1) medical students (MD, *n* = 407 (150 males), age 21.63 ± 2.06 yrs), (2) nursing students (RN, *n* = 155 (23 males), age 26.79 ± 8.94 yrs), and (3) medical laboratory diagnostics students (MLD, *n* = 150 (28 males), age 22.53 ± 7.17 yrs). Students were grouped in those three groups to compare whether there is any difference between different healthcare professions concerning personality dimensions and morningness-evenings orientation. The study was approved by the Ethical Committee of the Faculty of Medicine Osijek (Number 2158-61-07-15-77). The present study was conducted according to the Declaration of Helsinki and its amendments.

### 2.2. Questionnaires

Students were asked to fill in a questionnaire consisting of three parts. In the first part, they were asked about their socio-demographic and academic characteristics.

The second part consisted of the reduced morningness-eveningness questionnaire (rMEQ). The MEQ was developed by Horne and Östberg and is the most widely used morningness measure [4]. Adan and Almirall developed the reduced MEQ (rMEQ) [30]. This scale contains five items, and the correlation between the rMEQ and the MEQ ranges from satisfactory to excellent (0.69–0.90) [1]. The total rMEQ score, obtained by summing the scores of each question, ranges from 4 to 25, with higher rMEQ scores indicating a morningness preference. We used the cut-offs as suggested by Adan and Almirall [30] to divide our sample into three circadian groups (morning types, neither types and evening types). In this study, the questions of the rMEQ were drawn from a Croatian translation of the MEQ [31] previously used in similar research [8].

In the third part, students were questioned about their characteristic traits using the International Personality Item Pool Five-Factor questionnaire (IPIP Big-5). The IPIP was created by Goldberg for the development of advanced measures of personality traits and other individual differences [32]. We measured personality traits with the Croatian version of the questionnaire IPIP Big-Five with 50 items (short version) [33]. Participants were asked to read each of the 50 items and then rate how well they believed it described them on a 5-point rating scale ranging from 1 (very inaccurate) to 5 (very accurate) as in the original instrument [32]. This scale was used for several reasons: there is a scarcity of validated psychological instruments in Croatian when compared to larger countries; this instrument is open access and proved useful in similar research on students [16]; it contains 50 questions which makes it large enough to provide good internal validity, but is short enough to be useful in large cross-sectional studies; it has good psychometrical properties [34]. In the original American sample the Cronbach alpha scores were as follows: extraversion 0.87, agreeableness 0.82, conscientiousness 0.79, emotional stability 0.86, and intellect/imagination 0.84; the validated Croatian instrument had similar Cronbach alpha scores: 0.87, 0.79, 0.81, 0.88, and 0.79 [33].

### 2.3. Statistical Analyses

Cronbach alpha scores were calculated to assess the reliability of the instruments. The dimension reduction was performed using a principal component analysis (PCA). A Kolmogorov–Smirnov test was used to assess the normality of the data distribution. IPIP Big-Five and rMEQ variables deviated from a normal distribution, but due to the large sample size, it was possible to use parametric tests. Numerical data were described as means and standard deviations (SD). To compare the means of two or more independent groups, a *t*-test and one-way ANOVA tests were used, respectively. Effect sizes were calculated using the formulas suggested by Tomczak and Tomczak [35] and Fritz et al. [36]. Even though the distribution deviated from normal, the large sample size allowed for the use of analyses of covariance (ANCOVA) considering the total score of each dimension of the IPIP Big-Five and rMEQ score as a dependent variables and taking study majors as factor, while age was considered as a covariate to control for possible effects. The partial eta-squared (η_p_^2^) was obtained as a measure of effect size, and the observed statistical power for significant effects was >0.90. Correlation between variables was determined using the Pearson’s rank-order correlation coefficient. *p* < 0.05 was considered statistically significant, and all values were adjusted for multiple testing, according to Bonferroni. The analysis was conducted using SPSS software (ver. 16.0, SPSS Inc., Chicago, IL, USA).

## 3. Results

### 3.1. Reliability of IPIP 50 Big Five and rMEQ

The Cronbach alpha for the rMEQ in the total sample was 0.638. Detailed information on corrected item-total correlations, alpha if items deleted and factor loadings of the items of the Croatian rMEQ are presented in Table 1. Inter-item correlations of the items of the Croatian rMEQ are presented in Table 2.

Cronbach alphas for the subscales of the IPIP Big-5 were as follows: extraversion 0.82, agreeableness 0.73, conscientiousness 0.71, emotional stability 0.86, intelligence 0.77.

The results from the principal component analysis (PCA) showed a single factor solution of the rMEQ scale, Eigenvalue 2.308, that explained 46.17% of the variance. The Kaiser–Meyer–Olkin (KMO) test showed acceptable sampling adequacy (0.750) with an approximated Chi-Square 641.875. Bartlett’s test showed no redundancy (*p* < 0.001, df 10).

### 3.2. IPIP 50 Big-Five and rMEQ Related Differences between Different Majors

Several significant differences were found between the students of different majors (Table 3). There was a significant age difference between the groups (*p* < 0.001, one-way ANOVA, *F* (2) = 49.23, η^2^ = 0.12). Nursing students were significantly older than MD students (*p* < 0.001, *t*-test, *t* (558) = 10.93, Cohen’s d **=** 1.04), and MLD students (*p* < 0.001, *t*-test, *t* (301) = 4.56, Cohen’s d = 0.53). MLD students were significantly older than MD students (*p* < 0.001, *t*-test, *t* (555) = 2.29, Cohen’s d = 0.22).

As it can be seen in Table 3, RN students scored higher than MD students (*p* = 0.001, *t*-test, *t* (560) = 3.45, Cohen’s d = 0.33), and the MLD students (*p* < 0.001, *t*-test, *t* (303) = 5.24, Cohen’s d = 0.60) on the rMEQ and the MD students scored higher than the MLD students (*p* = 0.023, *t*-test, *t* (555) = 2.28, Cohen’s d = 0.22).

**Figure 1:**
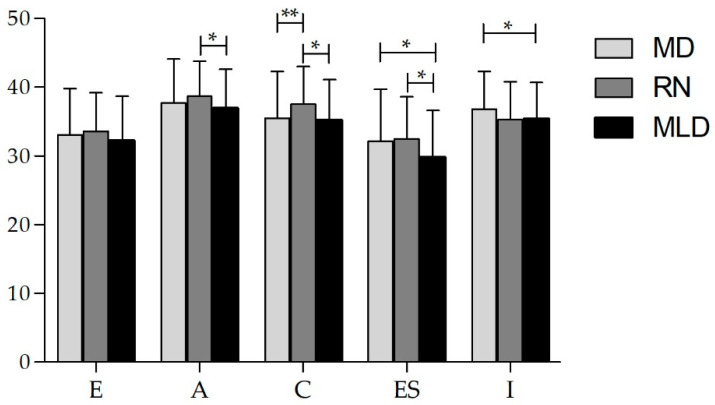
Personality dimensions of IPIP Big-Five (mean ± SD) within different majors (ANCOVA analysis, adjusted for age). E–extraversion; A–agreeableness; C–conscientiousness; ES–emotional stability; I–intellect; MD–medical students; RN–nursing students; MLD–medical laboratory diagnostics students; * *p* < 0.05; ** *p* < 0.001. *n* = 712.

MD students scored significantly lower than RN students on the conscientiousness subscale (*p* = 0.001, *t*-test, *t* (560) = 3.35, Cohen’s d = 0.32) but scored higher on the intellect subscale (*p* = 0.002, *t*-test, *t* (560) = 3.05, Cohen’s d = 0.29). MD students also scored significantly higher on both the intellect (*p* = 0.005, *t*-test, *t* (555) = 2.79, Cohen’s d = 0.27) and emotional stability subscales when compared with the MLD students (*p* = 0.001, *t*-test, *t* (555) = 3.36, Cohen’s d = 0.32). Nursing students scored significantly higher on the extraversion (*p* = 0.035, *t*-test, *t* (303) = 2.12, Cohen’s d = 0.24), agreeableness *(p* = 0.005, *t*-test, *t* (303) = 2.81, Cohen’s d = 0.32), conscientiousness (*p* = 0.001, *t*-test, *t* (303) = 3.51, Cohen’s d = 0.40) and the emotional stability scale (*p* < 0.001, *t*-test, *t* (303) = 3.57, Cohen’s d = 0.41) than the MLD students (Figure 1).

An ANCOVA showed that nursing students presented higher average scores in agreeableness (F_(2706)_ = 4.356; *p* = 0.013; η_p_^2^ = 0.012), and conscientiousness (F_(2706)_ = 7.428; *p* < 0.001; η_p_^2^ = 0.021), while MLD students had the lowest score in emotional stability (F_(2706)_ = 7.316; *p* < 0.001; η_p_^2^ = 0.02). MD students had the highest score in intellect dimension (F_(2706)_ = 3.575; *p* = 0.029; η_p_^2^ = 0.01). Post-hoc comparisons between students’ major groups for personality dimensions indicated that the RN students had a significantly higher agreeableness score when compared with MLD (2.04, *p* = 0.012). Furthermore, RN students had a significantly higher conscientiousness score when compared with the MD (2.29, *p* < 0.001) and MLD students (2.52, *p* = 0.002). MLD students had a significantly lower emotional stability score when compared with MD (−2.33, *p* = 0.002) and RN students (−2.80, *p* = 0.003). MD students had a significantly higher intellect score compared to MLD (1.31, *p* = 0.033).

The distribution of students’ circadian preferences was 86 in the morning type (12.1%), 406 for neither type (57.2%) and 218 in the evening type (30.7%). Table 4 shows the distribution of circadian preference (rMEQ) and descriptive statistics (mean ± standard deviation) between the different majors. There was a significant difference in circadian preferences (F_(2706)_ = 7.210; *p* < 0.001; η_p_^2^ = 0.02) between different majors while adjusting for age (ANCOVA). Post-hoc comparison between different majors and circadian preferences indicated that the MLD students had a significantly lower score when compared with MD (−0.96, *p* = 0.025) and RN students (−1.69, *p* = 0.001). There was no difference between MD and RN students (−0.73, *p* = 0.176) on circadian preference.

Gender-related differences can be seen in Tables S1 and S2 (Supplementary Material). There was no difference between gender and rMEQ (*t* (710) = 0.30, *p* = 0.764). Men’s lower scores in agreeableness were observed in the evening type group, while in the neither type and the morning type groups no significant differences between men and women were observed.

### 3.3. Relationship of IPIP 50 Big-Five and rMEQ

Correlations between the rMEQ and IPIP Big-Five can be seen in Table 5**.** Circadian preference positively correlated with age (r = 0.178, *p* < 0.01). Further, circadian preference positively correlated with some of the personality dimensions, i.e., conscientiousness (r = 0.232, *p* < 0.01), and emotional stability (r = 0.133, *p* < 0.01), which indicates that more morning-oriented students are more responsible and emotionally stable.

Table 6 shows descriptive statistics of the domains of the IPIP 50 Big-Five questionnaire according to morningness-eveningness orientation.

**Figure 2:**
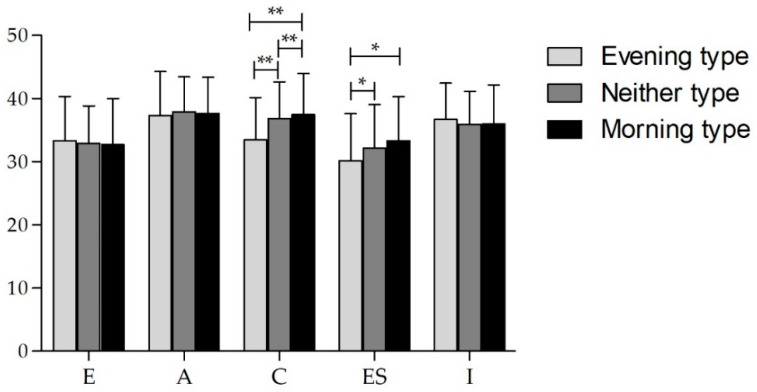
Personality dimensions of IPIP Big-Five (mean ± SD) and rMEQ interaction. E–extraversion; A–agreeableness; C–conscientiousness; ES-emotional stability; I–Intellect; * *p* < 0.005; ** *p* < 0.001. *n* = 712.

Morningness-eveningness orientation (rMEQ) presented significant differences in the ANCOVA for the dimensions conscientiousness (F_(2706)_ = 24.573; *p* < 0.0001; η_p_^2^ = 0.065) and emotional stability (F_(2706)_ = 8.703; *p* < 0.0001; η_p_^2^ = 0.024). Post-hoc comparisons between circadian typology groups indicated that the evening type had a significantly lower conscientiousness score when compared with neither type (−3.42, *p* < 0.0001) and morning type (−4.04, *p* < 0.0001). Also, evening type had a significantly lower emotional stability score when compared with neither type (−2.06, *p* = 0.002) and morning type (−3.29, *p* = 0.001). Higher morningness scores corresponded to higher scores of conscientiousness and emotional stability (Figure 2).

## 4. Discussion

This study utilized the IPIP 50 Big-Five model and the rMEQ to examine links between personality and individual differences in circadian rhythm among three groups of students, based on their major, as well as the psychometric reliability of the rMEQ and IPIP Big-Five in the population of students from biomedical studies.

The psychometric properties of the Croatian rMEQ in this study sample are modest (Cronbach alpha 0.638). It should be noted, however, that the results are not drastically different from the scales translated into other languages, where the alpha is usually around 0.7 [37], which is decent considering the short length of the scale. One possible reason can be found when observing inter-item correlations and factor loadings. It can be observed that Item 3 correlated the worst with the other items (Table 2) and it had the lowest factor loading (0.388, Table 1). The English formulation of the item is: “At what time in the evening do you feel tired and as a result in need of sleep?” It is possible that biomedical students have a habit of going to bed later than their bodies would normally require, and that the force of habit makes them biased when answering this item. This might indicate that this instrument is not ideal for this population of students, and more studies should be therefore performed in the general population. To the authors’ knowledge, no other research has been done in Croatia using the rMEQ. A validation study of the Slovenian MEQ showed that the results are similar to the findings from previous studies. Even with a much smaller number of participants in the Slovenian study, MEQ was useful for measuring morningness-eveningness orientation [38]. Circadian preferences correlated with other psychological constructs in several studies focused on Eysenck’s personality dimensions [12,27,39]. The IPIP Big-five is a reliable measure that shows high sensitivity in determining students’ personality dimensions, and the internal reliability for the present sample is high (Cronbach’s α = 0.77).

The distribution between chronotypes is similar to that found in literature [5,6], with 52.7–63% of our students belonging to neither type. However, we found differences between the majors, with RN students having a similar distribution of morning and evening types, and a larger proportion of students adhering to neither type. In contrast, MD and MLD students had one third to two-fifths of students adhering to evening type, compared to about 10% of students adhering to morning types. A possible explanation is that RN students are older than MD and MLD students, so they showed a tendency to move from evening to morning type. Age might have some impact on the morningness level of RN students, since it has been shown that people become more morning-oriented as they age [40]. In our study, RN students were 1.02 standard deviations older than MD students which represents a difference of 5 years. Statistical significance of the correlation between morningness and age means that those who were older scored higher on morningness. RN students were the oldest and had the highest score on morningness in the study, which is similar to the results of Muro et al. [9]. Due to the fact that nurses in Croatia work alternating day and night 12-h shifts, the greater percentage of neither type rhythm might be a favorable predisposition for their future professional working schedule, whereas they could have a greater problem adapting to these alternating shifts if they belonged to one of the extreme circadian groups. For instance, it has been shown that evening-oriented shift nurses have poorer subjective sleep quality, which can cause issues as daytime sleepiness [41], therefore leading to chronic fatigue. Furthermore, since the regular working shifts of physicians are 8-h shifts starting in the morning, this could be a reason for struggle and poorer working performance in their future professional life. This could be also applied to medical laboratory professionals. However, greater eveningness might be favorable for future physicians in the perspective of 24-h shifts, which physicians work several times monthly, where a more morning-oriented person probably might feel too tired to properly deal with medical emergencies with enough mental concentration.

The IPIP Big-Five results showed that MD students scored higher on emotional stability and intellect than the MLD students, so they tended to be calmer and more relaxed compared to MLD students. They do not react with intense emotions and have intellectual and artistic interests [14]. The results showed that MLD students had low extraversion scores, which might be an indicator of emotional vulnerability to anxiety disorders [42]. When associated with low emotional stability, this might predict obsessive-compulsive traits, an aspect more commonly seen in evening types [12]. In this study, MLD students had the lowest score on extraversion and emotional stability, and were more evening-oriented, so they might be at risk for affective disorders. On the other hand, agreeableness presents self-control regarding disciplined aspirations toward goals and strict adherence to personal principles [43]. A high score on agreeableness is marked by kindness, caring and sympathy [14]. These are traits often desired in nursing professionals and that is in concordance with our results where RN students had the highest scores. Lastly, conscientiousness is a significant predictor of various aspects of work performance [11]. RN students had the highest score on conscientiousness, which implies that they could be more reliable, well-organized, responsible and hard-working [14].

Authors have suggested a link between agreeableness, conscientiousness and morningness. The possible explanation of the connection between personality and morningness may lay in neurochemistry, as serotonin is strongly involved in the modulation of circadian preferences and serotonergic differences influence both circadian preferences and personality [28]. Another possible explanation is that circadian preference and the Big-Five model of personality have a genetic basis [27,28]. Results showed that conscientious students were more morning-oriented. This result is relevant, even though it might be known that conscientious students are probably more likely to spend more time studying. Since biomedical disciplines have a very demanding workload, students are often forced to study at night, which can have detrimental consequences on individuals naturally inclined to go to bed early. The authors’ previous research found that almost 30% of MD students showed excessive sleepiness and only 21.7% of students slept 8 h or more at night, which is the amount of sleep recommended to adult individuals by the WHO to recover from physical and psychological fatigue [41,44]. On the other hand, emotionally unstable students were more evening-oriented, which was also found in certain studies [19,20]. It is interesting that MD students were shown to be more stable than RN students, especially since greater morningness was found to correlate to emotional stability and RN students were more morning-oriented compared to MD students. This may also indicate that in the case of MD students, the evening orientation could be due to circadian misalignment. It is possible that MD students tend to be more evening types than their peers, and since their workload is greater, this can also impact their wellbeing. Morningness-eveningness orientation should be considered as a significant risk factor for burnout, as observed in evening types [45]. Furthermore, evening-oriented individuals tend to have more depressive symptoms, as well as cognitive and behavioral problems, experiencing hyperactivity, attention deficit and tending towards impulsivity [21,46]. Circadian preferences are associated with emotional stability and affective disorders, which is particularly evident in medical students [16]. In the present study, it was MLD students who had such results of low emotional stability and evening orientation. In a prior research at our school, more than half of students showed some level of depressive symptoms [16]; we find these results alarming. Sleep deprivation caused in part by early classes and nights spent studying might have a causative role. It is considered that individual differences in serotonergic function are a primary source of stability as a trait [19].

The association of chronotype and personality in a broad social context might help to explain social influences on circadian preference and human personality traits more clearly [47]. The effects of circadian preferences on cognitive function may be small but are worthy of further consideration. Personality characteristics of the evening-type and early-morning-type of people entail different vulnerability to risky and addictive behaviors such as smoking, drugs and alcohol [48]. It is important to mention that age is a variable that significantly influences circadian preferences [19], which is observed in our study.

Our study showed gender differences in personality dimensions, in agreement with other research [11,18,49,50]; female students scored lower than males on the emotional stability and higher on the agreeableness scale. However, gender-related differences were not found in the extraversion and conscientiousness scale, and this is in accordance with the results obtained by Prat and Adan [18]. Personality dimensions are gender-related and could be used to assess risk or protect mental health based on gender [39]. In the literature, gender analyses of circadian preferences are inconsistent. In this study, no difference between gender and circadian preference was found, as was the case in the study of Prat and Adan [18], even though differences have been found in a study on adolescents [8], in a study with a much larger sample [51] or with the use of meta-analytic procedures [52].

Our study has several limitations. First, we assessed the relationship between university students of different majors and IPIP50 Big-five model and rMEQ. These subjects most likely have different characteristics compared to general population samples, such as particular cultural backgrounds or socio-economic status, as well as some control over their daily schedules. Furthermore, the sample was biased toward women, which is a result from the student body consisting dominantly of female students. Furthermore, the sample was biased toward MD students because they represent the majority of enrolled students. Students were not tested on other variables which might have proven useful, such as sleep quality, sleep duration, average time spent studying, depression, anxiety or perfectionism, and these variables should be assessed in future research.

## 5. Conclusions

This research contributes to the knowledge about the relationship between circadian preferences and personality among students of health-related university majors. This is especially relevant to Croatia, where little research has been done on the topic of circadian typology. Chronobiology is often an ignored topic by medical professionals. However, working at top performance often comes as a privilege, and many who work in the medical branches cannot afford it. For a vast number of medical professionals, the medical systems, with inflexible shifts and unexpected emergency duty calls, are a constant source of frustration and anxiety, thereby undermining their professional success [21,41]. Just as chronobiology is mostly not acknowledged in the professional life of physicians, medical laboratory professionals and nurses, it also rarely becomes appreciated as a source of potential threat for the mental and metabolic health of patients. The results indicated that MD students are the most imaginative, while RN students were the most caring and responsible students. Further, RN students were the most morning-oriented, while MLD students were more shy and evening-oriented than other groups. According to the present study, MLD students might be at risk of developing affective disorders. These results suggest that circadian preference and personality traits are essential components of the well-being of many medical professionals [53]. With this research, we would like to raise awareness about common personality traits and adherence to certain circadian preferences in medical students to have data for later comparisons and considerations about the timetables for classes, i.e., if most of the students are evening types, classes should be put later in the day, and not in the early morning. Early classes may lead to sleep deprivation and affective disorders, already highly prevalent in this population [16]. Data on the chronotypes of future medical professionals are appealing because they could be compared to those of skilled medical professionals, after they start working shifts, to see if their chronotypes change after they enter the workforce. It is possible to investigate the relationship between personality dimensions among skilled medical professionals to see how it differs from the data obtained in this research on the student population.

Psychometric analysis of the Croatian version of the rMEQ showed mediocre results, and additional tests are warranted to assess its reliability in the general population. Future research should compare the dimensions of personality dimensions and morningness-eveningness orientation in employees who completed these different majors.

## Figures and Tables

**Table 1:** Item means and standard deviations, corrected item-total correlations, alpha if items deleted and factor loadings of the items of the Croatian rMEQ (*n* = 712).

Item	Mean ± SD	Corrected Item-Total Correlations	Alpha If Item Deleted	Factor Loadings
1. Considering only your own “feeling best” rhythm, at what time would you get up if you were entirely free to plan your day?				
	2.87 ± 0.87	0.502	0.557	0.743
2. During the first half-hour after having woken in the morning, how tired do you feel?				
	2.29 ± 0.76	0.357	0.611	0.607
3. At what time in the evening do you feel tired and as a result in need of sleep?				
	2.76 ± 1.46	0.244	0.673	0.388
4. At what time of the day do you think that you reach your “feeling best” peak?				
	2.70 ± 0.79	0.536	0.554	0.755
5. One hears about “morning” and “evening” types of people. Which one of these do you consider yourself to be?				
	2.69 ± 1.83	0.577	0.490	0.816

**Table 2:** Inter-item correlations of the items of the Croatian rMEQ (*n* = 712).

Variable	Item 2	Item 3	Item 4	Item 5
Item 1	0.360	0.164	0.397	0.477
Item 2		0.073	0.295	0.338
Item 3			0.182	0.251
Item 4				0.543

**Table 3:** Descriptive statistics of the reduced Morningness-eveningness questionnaire (rMEQ) and the domains of the IPIP 50 Big-Five questionnaire and the differences between the groups (*n* = 712).

Variable	MD	RN	MLD	All Students	ANOVA *		
	*n* = 407	*n* = 155	*n* = 150				
	Mean ± SD	Mean ± SD	Mean ± SD	Mean ± SD	F	*p*-Value	η^2^
Age	21.63 ± 2.1	26.79 ± 8.94	22.53 ± 7.17	22.93 ± 5.88	49.23	<0.001	0.12
rMEQ	13.23 ± 4.1	14.47 ± 3.13	12.35 ± 3.89	13.32 ± 3.89	11.84	<0.001	0.03
Extraversion	33.14 ± 6.69	33.62 ± 5.56	32.15 ± 6.52	33.04 ± 6.44	2.12	0.121	0.01
Agreeableness	37.66 ± 6.35	38.68 ± 5.12	36.93 ± 5.72	37.73 ± 5.99	3.32	0.037	0.01
Conscientiousness	35.49 ± 6.75	37.53 ± 5.49	35.24 ± 5.88	35.89 ± 6.37	6.78	0.001	0.02
Emotional Stability	32.16 ± 7.49	32.45 ± 6.06	29.81 ± 6.83	31.73 ± 7.13	7.09	0.001	0.02
Intellect	36.84 ± 5.46	35.27 ± 5.49	35.39 ± 5.28	36.19 ± 5.47	6.77	0.001	0.02

MD–medical students; RN–nursing students; MLD–medical laboratory diagnostics students; SD–standard deviation; * one-way ANOVA df 2, 711.

**Table 4:** Distribution and descriptive statistics of morningness-eveningness orientation (rMEQ) according to gender and within the different majors (*n* = 712).

Variable	Morning Type		Neither Type		Evening Type	
	*n* (%)	Mean ± SD	*n* (%)	Mean ± SD	*n* (%)	Mean ± SD
MD (*n* = 406)	48 (11.8)	20.1 ± 3.4	223 (54.9)	14.5 ± 1.1	135 (33.3)	8.9 ± 1.8
RN (*n* = 155)	26 (16.8)	19.6 ± 1.7	104 (67.1)	14.4 ± 1.1	25 (16.1)	10.2 ± 0.9
MLD (*n* = 149)	12 (8.1)	19.4 ± 0.8	79 (53)	14.3 ± 1.2	58 (38.9)	8.4 ± 2.0

MD–medical students; RN–nursing students; MLD–medical laboratory diagnostics students; SD–standard deviation.

**Table 5:** Correlations (Pearson’s r) of age, reduced morningness-eveningness (rMEQ), and the domains of the IPIP 50 Big-Five questionnaire (*n* = 712).

Variable	rMEQ	Extraversion	Agreeableness	Conscientiousness	Emotional Stability	Intellect
Age	0.178 *	−0.032	−0.040	0.022	−0.006	−0.179 *
rMEQ		−0.033	0.012	0.232 *	0.133 *	−0.031
Extraversion			0.265 *	0.160 *	0.237 *	0.289 *
Agreeableness				0.317 *	0.012	0.308 *
Conscientiousness					0.280 *	0.224 *
Emotional Stability						0.067

* *p* < 0.01; Pearson’s r.

**Table 6:** Descriptive statistics (mean ± SD) of the IPIP 50 Big-Five questionnaire domains according to the reduced morningness-eveningness questionnaire (rMEQ).

IPIP 50	rMEQ			ANOVA *		
	Morning Type	Neither Type	Evening Type	F	*p*-Value	η^2^
	*n* = 86	*n* = 406	*n* = 218			
Extraversion	32.7 ± 7.3	32.9 ± 5.9	33.4 ± 6.9	0.44	0.643	<0.01
Agreeableness	37.7 ± 5.7	37.9 ± 5.5	37.4 ± 6.9	0.67	0.499	<0.01
Conscientiousness	37.4 ± 6.5	36.8 ± 5.8	33.5 ± 6.7	24.39	<0.001	0.06
Emotional Stability	33.4 ± 6.9	32.2 ± 6.9	30.2 ± 7.5	8.31	<0.001	0.02
Intellect	36.0 ± 6.1	35.9 ± 5.2	36.7 ± 5.7	1.42	0.241	<0.01

SD-standard deviation; * one-way ANOVA df 2, 711.

## Supplementary Materials

The following are available online at https://www.mdpi.com/1660-4601/17/13/4794/s1, Table S1: Distribution and descriptive statistics of morningness-eveningness orientation (rMEQ) according to gender, Table S2: Descriptive statistics of the reduced morningness-eveningness (rMEQ) questionnaire and the domains of the IPIP 50 Big-Five questionnaire and the gender-related differences (*n* = 712).
