# Supplementary material for: Circadian Typology and Personality Dimensions of Croatian Students of Health-Related University Majors
Source: Int J Environ Res Public Health. 2020 Jul 3;17(13):4794. doi: 10.3390/ijerph17134794 (PMC7370063; doi:10.3390/ijerph17134794)
Supplement: Supplementary file 1 [file ijerph-17-04794-s001.pdf]

Table 1. Distribution and descriptive statistics of morningness-eveningness orientation (rMEQ) according to gender.

Variable	Morning Type		Neither Type		Evening Type	
	n (%)	Mean ± SD	*n* (%)	Mean ± SD	*n* (%)	Mean ± SD
Male (*n* = 199)	26 (13)	20.7 ± 4.3	104 (52.3)	14.4 ± 1.2	69 (34.7)	9.0 ± 1.9
Female (*n* = 511)	60 (11.7)	19.5 ± 1.5	302 (59.1)	14.4 ± 1.1	149 (29.2)	8.9 ± 1.9

SD—standard deviation.

Table S2. Descriptive statistics of the reduced Morningness-Eveningness (rMEQ) questionnaire and the domains of the IPIP 50 Big-Five questionnaire and the gender-related differences (*n* = 712).

Variable	Male	Female	ANOVA *		
	Mean ± SD	Mean ± SD	F	*p*-Value	η^2
Age	21.73 ± 3.95	23.4 ± 6.43	11.92	0.001	0.02
rMEQ	13.24 ± 4.39	13.34 ± 2.68	0.09	0.764	<0.001
Extraversion	33.62 ± 6.45	32.81 ± 6.43	2.30	0.130	<0.001
Agreeableness	35.44 ± 6.04	38.63 ± 5.74	43.17	<0.001	0.06
Conscientiousness	35.85 ± 6.06	35.90 ± 6.49	0.01	0.917	<0.001
Emotional stability	33.89 ± 7.43	30.87 ± 6.83	26.88	<0.001	0.04
Intellect	37.22 ± 5.67	35.79 ± 5.34	9.93	0.002	0.01

SD—standard deviation; * One-way ANOVA df 2.
